# Illness perception and its relationship with nursing care behaviors in hemodialysis patients: A cross-sectional descriptive study

**DOI:** 10.1097/MD.0000000000050481

**Published:** 2026-09-04

**Authors:** Özlem Bulantekin Duzalan, Elif Bulbul, Selda Selimoglu Namoglu

**Affiliations:** a Nursing Department, Faculty of Health Science, Alanya Alaaddin Keykubat University, Antalya, Turkiye; b Hamidiye Faculty of Nursing, University of Health Sciences Turkey, Istanbul, Turkiye; c Sultangazi Dialysis Centers, Istanbul, Turkiye.

**Keywords:** hemodialysis, illness perception, nursing care, nursing caring behavior

## Abstract

This study aimed to evaluate the relationship between hemodialysis patients’ illness perception and nursing care behavior. This cross-sectional descriptive study was conducted between January and July 2022 at a high-volume outpatient hemodialysis center with 40 dialysis machines. Consecutive (convenience) sampling was used, and 223 patients undergoing hemodialysis were included. Data were collected using a sociodemographic form, the Brief Illness Perception Questionnaire (BIPQ), and the Caring Behaviors Inventory (CBI)-24. Independent samples *t*-test, Analysis of variance, Pearson correlation analysis, and multiple linear regression analysis were applied. Total scores were 51.08 ± 5.58 for the BIPQ and 4.82 ± 0.45 for the CBI-24, and a statistically significant positive correlation was found between them (*r* = 0.214; *P* = .001). A significant negative correlation was found between the CBI-24 assurance subscale and the cognitive subscale of the BIPQ (*r* = –0.152; *P* = .023). Significant positive correlations were observed between the knowledge and skill subscale and the emotional (*r* = 0.234; *P* = .001) and cognitive (*r* = 0.145; *P* = .030) subscales, between the being respectful and emotional subscales (*r* = 0.192; *P* = .004), and between the connectedness and emotional subscales (*r* = 0.275; *P* = .000). Education status, access type, having care help, years of dialysis treatment, and BIPQ explained 24.4% of the variance (*F* = 9.887; *R*^2^ = 0.244; *P* = .000). As the duration of illness increased, patients’ illness perception was negatively affected. These findings highlight the importance of individualized and holistic nursing interventions to improve illness perception and strengthen treatment adherence in hemodialysis patients.

## 1. Introduction

Chronic kidney disease is a major public health problem worldwide and in Turkey. Although disease progression can often be prevented or delayed when detected early, low awareness and delayed diagnosis limit these opportunities in many patients. This progressive condition affects more than 10% of the global population, corresponding to more than 800 million people. As chronic kidney disease progresses to end-stage kidney disease, kidney replacement therapy is necessary, with maintenance hemodialysis being the most commonly used treatment modality worldwide. Despite its life-sustaining role, maintenance hemodialysis is associated with substantial physical, psychological, and social burdens that adversely affect patients’ quality of life and overall well-being.^[1]^

In the treatment of end-stage kidney disease, hemodialysis remains the most widely used renal replacement therapy. It affects daily life in many ways, including diet, fluid intake, multiple therapeutic interventions, and self-management, necessitating a comprehensive care plan. Hemodialysis patients are faced with a heavy load of interventions, which affect not only their physical and mental health but also their families, lifestyle, ability to work, and quality of life. In addition to these physical challenges, patients undergoing maintenance hemodialysis frequently experience psychological distress, including anxiety, depression, uncertainty, and reduced emotional well-being, all of which may influence their adaptation to treatment and disease management.^[2,3]^

Illness perception refers to an individual’s perspective, focusing on their experience and understanding of their condition. Chronic illnesses provide insights into how health behaviors can be maintained.^[4,5]^ Individuals with acute and chronic illnesses develop their own belief systems to make sense of their medical conditions and create coping strategies. These belief systems, also known as illness perceptions, have been theorized within Leventhal Common-Sense Model of Self-Regulation. Illness perceptions are characterized by features such as the identity of the illness, its perceived cause, whether it is acute, chronic, or cyclical, the perceived severity of the condition, beliefs about treatment, and the emotional responses it triggers, including anxiety and worry.^[6,7]^ According to this theoretical framework, illness perceptions influence coping behaviors, treatment adherence, emotional adjustment, and health outcomes. Therefore, understanding illness perception is particularly important among patients receiving maintenance hemodialysis who must adapt to lifelong treatment, complex self-management requirements, and repeated therapeutic interventions. Understanding how these patients perceive their illness can inform tailored nursing strategies to enhance adherence and psychological well-being.^[3,4]^

Illness perception is not static but evolves throughout the disease trajectory and is influenced by patients’ experiences with symptoms, treatment, healthcare professionals, and social support. In hemodialysis patients, perceptions of maladaptive illness have been associated with poorer treatment adherence, lower quality of life, and less effective coping with the demands of long-term therapy.^[4,5]^ Consequently, identifying factors associated with illness perception is important for improving patient-centered care in this population.

A previous study by Delmas et al reported that patients receiving maintenance hemodialysis generally perceived nursing caring behaviors positively and highlighted the importance of the nurse–patient relationship in this population. The authors compared patients’ and nurses’ perceptions of caring behaviors and found that patients tended to evaluate nursing care more positively than nurses themselves. However, their study focused on differences in perceived caring behaviors and did not examine whether illness perception was associated with patients’ evaluations of nursing care. Therefore, the relationship between illness perception and perceived nursing care behaviors in patients undergoing maintenance hemodialysis remains unclear.^[8]^ The present study aimed to examine this relationship in a sample of patients undergoing maintenance hemodialysis. Two basic abilities are necessary for care behavior. The first is emotional intelligence, reliability, and sensory ability. The second category is related to technical skills, therapeutic interventions, and crisis stress management.^[9]^ Maintenance hemodialysis requires patients to spend approximately 4 hours per session, 3 times weekly, in continuous contact with nursing staff, which provides a unique therapeutic environment in which nurses can establish sustained interpersonal relationships with patients. This prolonged interaction allows nurses to not only deliver technical care but also assess patients’ cognitive and emotional responses to their illness, provide individualized education, emotional support, and counseling, and promote adaptive coping. Consequently, nursing care behaviors may play an important role in shaping patients’ illness perceptions and facilitating successful adaptation to long-term treatment.^[4]^ Hemodialysis is a treatment method that can make diet and fluid restriction difficult. Nurses also play a key role in this regard.^[10]^ In particular, nurses must assess patients’ needs and provide each individual with specific, holistic care. Maintenance hemodialysis requires patients to spend approximately 4 hours per session, 3 times weekly, in direct contact with nursing staff: a sustained therapeutic encounter qualitatively distinct from most acute care nursing interactions. This prolonged proximity creates a structured opportunity for nurses to assess and respond to patients’ cognitive and emotional representations of their illness, rendering the dialysis unit a particularly relevant setting in which to examine the relationship between illness perception and nursing care behavior.^[2,8,11]^

Previous studies have primarily focused on the associations between illness perception and clinical outcomes, such as treatment adherence, self-management, psychological distress, and quality of life among patients receiving hemodialysis. Similarly, nursing care behaviors have been examined in relation to patient satisfaction, quality of care, and clinical outcomes. However, evidence regarding the relationship between patients’ illness perceptions and perceptions of nursing care behaviors remains limited.^[3,12,13]^ Clarifying this relationship may provide evidence to support nursing interventions aimed at improving psychological adaptation and patient-centered care in maintenance hemodialysis settings.

Therefore, this study aimed to evaluate the relationship between illness perception and perceived nursing care behaviors among patients undergoing maintenance hemodialysis. Specifically, this study sought to answer the following research questions:

- What are the illness perception levels of patients receiving maintenance hemodialysis?
- What are the perceived nursing caring behavior levels of patients receiving maintenance hemodialysis?
- Is there a significant relationship between illness perception and perceived nursing care behaviors among patients undergoing maintenance hemodialysis?

## 2. Methods

### 2.1. Design

A cross-sectional descriptive correlational design was employed, as this approach is appropriate for examining the associations between 2 continuous variables at a single point in time without inferring causality. This design enables the simultaneous assessment of illness perception and nursing care behavior within the dialysis setting and is consistent with prior methodological approaches in renal nursing research. The study was reported in accordance with the Strengthening the Reporting of Observational Studies in Epidemiology statement for cross-sectional studies.

### 2.2. Data collection

Data were collected between January and July 2022 from patients receiving treatment at a single dialysis center. There were a total of 40 dialysis machines at the center, and on average, 3 dialysis sessions were conducted each day. In addition, 5 nurses, 10 dialysis technicians, and 1 medical specialist worked at the center.

Inclusion criteria for the research were being aged 18 years or older, receiving chronic hemodialysis treatment for longer than 6 months, having no auditory or visual impediments, no psychiatric illness, Alzheimer disease or dementia, and voluntary participation in communication and in the study.

A sample size equation for a known population was used to calculate the required sample size. At the time of data collection, 336 patients were undergoing dialysis. The sample size was determined using the formula n = (N.*t*^2^.*p*.*q*)/ *d*^2^(N − 1) + *t*^2^.*p*.*q*, where N is the population size (336), *t* is the standard normal deviation corresponding to a 95% confidence level (*t* = 1.96), *p* is the estimated proportion of attributes present in the population (*p* = .50), *q* = 1 − *p (q* = 0.50), and *d* is the margin of error (*d* = 0.05). Using these parameters, the required sample size was calculated as 223. A consecutive sampling (non-probability) method was employed, and data collection was terminated once this number was reached.

Eligible patients who attended the dialysis unit during the study period were consecutively invited to participate until the required sample size was achieved. All questionnaires were collected through face-to-face interviews conducted by trained researchers in a private dialysis unit. Participants were informed that their responses would remain confidential, would be anonymized before analysis, and would not influence the care they received or disclosed to the dialysis staff responsible for their treatment. Data were included consecutively until a target sample size of 223 was reached; therefore, there were no missing data requiring imputation or exclusion. All data collectors were trained in standardized procedures to ensure consistency in questionnaire administration, minimize interviewer bias, and recognize signs of participant discomfort during data collection. They were also blinded to the study hypotheses to reduce potential observer bias.

### 2.3. Data collection instruments

A Personal Information Form, the Brief Illness Perception Questionnaire (BIPQ), and the Caring Behaviors Inventory (CBI)-24 were used as data collection instruments. In the present study, data collection instruments were administered through face-to-face interviews using a standardized protocol for all participants.

#### 2.3.1. Sociodemographic data form

The Personal Information Form was developed by the researchers by scanning sources relevant to this topic. It consisted of 10 questions on age, sex, marital status, education status, employment status, economic status, whether they had anyone helping with care, vein route type, and duration of hemodialysis treatment.

#### 2.3.2. BIPQ

The BIPQ was developed by Broadbent et al in 2006.^[14]^ Validity and reliability were tested for Turkish by Karataş, Özen, and Kutlutürkan on patients with cancer, and its Cronbach alpha coefficient was 0.85.^[15]^ The Turkish version of the questionnaire consists of 7 items, plus 1 added item asking about causative factors. The 7 items other than the causative factors were scored on a 0 to 10 Likert-type scale. The questionnaire consists of 2 subscales: cognitive representations of illness and emotional representations of illness. Cognitive representations of illness consisted of 2 items on personal control, 3 on treatment control, and 6 on illness comprehensibility. The emotional representations of illness consisted of 1 item on the result, 4 on illness identity, 5 on worry, and 7 on emotional status. Along with these items, there was a final section with an open-ended question asking what were thought to be the 3 most important reasons for the illness. In evaluating the scale, the calculation of the score of the whole scale shows the extent to which the illness threatens or calms the patient. To calculate the scale score, items 2, 3 and 6 were scored in reverse and added to the scores of items 1, 4, 5, and 7. A high score indicates that the illness is a greater threat to the patient.^[15]^ In the present hemodialysis sample, internal consistency was confirmed with a Cronbach alpha of 0.83.

#### 2.3.3. CBI-24

The 42-item inventory, suitable for diagnostics by both patients and nurses, was reorganized by Wu et al (2006), with 24 items and 4 subscales.^[16]^ This scale was designed to evaluate the nursing care process. It is applied either by face-to-face or telephone interviews between the researcher and the patients, or by the patients completing it themselves. Kurşun and Kanan (2012) tested its validity and reliability in Turkish.^[17]^ The CBI consists of 24 items in 4 groups: assurance, knowledge and skill, being respectful, and connectedness. For the responses, a 6-way Likert-type scale was used, with 1 = never, 2 = almost never, 3 = sometimes, 4 = often, 5 = mostly, and 6 = always. In calculating the scale scores, the total scale score is obtained by adding together the scores of the 24 items and dividing by 24, resulting in a scale score between 1 and 6. To obtain subscale scores, the scores of the items in each subscale were added together, and the total was divided by the number of items in the subscale, giving a score between 1 and 6. Higher scores on the subscales and total scale indicate that patients perceive their levels of caring quality to be higher. A high score on the scale showed a more positive perception of care quality. In the present hemodialysis sample, internal consistency was confirmed with a Cronbach alpha of 0.93.

### 2.4. Statistical analysis

The statistical package Statistical Package for the Social Sciences 25.0 (SPSS Inc.) was used to evaluate the data. For descriptive variables, the mean ± standard deviation was calculated, and for nominal variables, the frequency and percentage rates were calculated. The normality of continuous variables was assessed using the Kolmogorov–Smirnov test. As the data showed a normal distribution, the independent samples *t*-test and 1-way analysis of variance were used to compare the total scores of the scales according to descriptive characteristics. When a significant difference was found in the analysis of variance, the least significant difference post hoc test was performed for multiple comparisons. The correlation between 2 continuous variables was examined using Pearson correlation analysis. The factors affecting CBI-24 were determined using multiple linear regression analysis. Multicollinearity among the independent variables included in the regression model was evaluated using the variance inflation factor and tolerance statistics before model construction. The level of statistical significance was set at *P* < .05.

### 2.5. Ethical considerations

In order to conduct this research, ethical approval was obtained from the Health Sciences University Hamidiye Non-Interventional Clinical Research Ethics Committee (Approval No: E-46418926-050.01.04-75040; approval date: November 3, 2021) before the commencement of data collection. Written informed consent was obtained from all participants prior to their participation. Participants were informed that their participation was entirely voluntary, that they could withdraw from the study at any time without providing a reason, and that their decision would not affect the dialysis treatment or nursing care they received. All questionnaires were anonymized prior to statistical analysis, and no personally identifiable information was recorded. Individual responses were accessible only to the research team and were not shared with the dialysis nurses or other clinical staff. Because the questionnaires included items related to illness perception and the evaluation of nursing care, participants were informed that some questions might evoke emotional reactions. Data collectors were trained to recognize signs of emotional discomfort and to discontinue the interview, if necessary. None of the participants required discontinuation or psychological referral during data collection. This study was conducted in accordance with the principles of the Declaration of Helsinki. The authors declare that they have no conflicts of interest. This research received no specific grants from any funding agency in the public, commercial, or not-for-profit sectors.

## 3. Results

A total of 223 patients were included in this study. Table 1 shows the participants’ sociodemographic characteristics. The mean age of the participants was 53.12 ± 14.04 years, 53.4% were male, 39.5% had a primary level education, 63.7% were married, 30.0% were working, and 66.4% had an income equal to expenses. The mean number of years of dialysis treatment was 5.70 ± 4.11 years, 72.6% had an arteriovenous fistula (AVF), 78.5% had a chronic illness, 78.5% regularly used medication, and 50.2% had someone to support their care (Table 1). The individuals’ mean total score was 4.82 ± 0.45 (95% confidence interval [CI]: 4.76–4.88) for the CBI-24 and 51.08 ± 5.58 (95% CI: 50.3–51.8) for the BIPQ. The mean CBI-24 subscale scores were 4.65 ± 0.53 (95% CI: 4.73–4.75) for assurance, 4.87 ± 0.82 (95% CI: 4.59–4.73) for knowledge and skill, 4.86 ± 0.77 (95% CI: 4.76–4.95) for being respectful, and 4.98 ± 0.78 (95% CI: 4.88–5.08) for connectedness. The mean BIPQ subscale scores were 32.77 ± 3.65 (95% CI: 32.8–33.2) for Emotional Representations of Illness and 18.31 ± 3.88 (95% CI: 17.8–18.8) for Cognitive Representations of Illness (Table 2).

**Table 1:** Distribution of patients by sociodemographic and clinical characteristics (N = 223).

	n	%
Age*	53.12 ± 14.04	21–82
Gender		
Female	104	46.60
Male	119	53.40
Marital status		
Married	142	63.70
Single/widowed	81	36.30
Education level		
Basic literacy	36	16.10
Primary school	88	39.50
Middle school	85	38.10
University	14	6.30
Employment		
Yes	67	30.00
No	156	70.00
Economy		
Income < expenditure	28	12.60
Income = expenditure	148	66.40
Income > expenditure	47	21.10
HD vintage*	5.70 ± 4.11	0.5–180
Vascular access		
AVF	162	72.60
Permanent catheter	51	22.90
Temporary catheter	10	4.50
Chronic illness		
No	48	21.50
Yes	175	78.50
Regular medication use		
Yes	175	78.50
No	28	12.60
Sometimes	20	9.00
Help with care		
Yes	112	50.20
No	111	49.80

AVF = arteriovenous fistula, HD = hemodialysis, N/n = number of patients, SD = standard deviation.

* Mean ± SD (Minimum–Maximum).

**Table 2:** Total score means of BIPQ and CBI-24 and their subscales.

	Mean ± SD	Min–Max	95.0% CI	
			Lower Bound	Upper Bound
BIPQ total score	51.08 ± 5.58	36.00–65.00	50.3	51.8
Emotional representations of illness	32.77 ± 3.65	21.00–40.00	32.8	33.2
Cognitive representations of illness	18.31 ± 3.88	20.00–27.00	17.8	18.8
CBI-24 total score	4.82 ± 0.45	3.04–5.58	4.76	4.88
Assurance	4.65 ± 0.53	2.88–5.88	4.59	4.73
Knowledge & skill	4.87 ± 0.82	2.00–6.00	4.76	4.98
Being respectful	4.86 ± 0.77	2.83–6.00	4.76	4.95
Connectedness	4.98 ± 0.78	2.60–6.00	4.88	5.08

BIPQ = Brief Illness Perception Questionnaire, CBI-24 = Caring Behaviors Inventory-24, CI = confidence interval, SD = standard deviation.

It was found to be statistically significant that patients included in the research who were working, had a university-level education, and whose income and expenditure were equal had higher CBI-24 total score means (*P* < .05). It was concluded that the care behaviors of patients with vein access route AVF and those with care support showed a statistically significant difference (*P* < .05). The illness perception of female patients was higher than that of male patients (*P* < .05). A weak positive correlation was found between dialysis vintage and the BIPQ total score (*r* = 0.154, 95% CI: 0.023–0.280, *P* = .021) and a moderate positive correlation between dialysis vintage and the CBI-24 total score (*r* = 0.286, 95% CI: 0.161–0.402, *P* < .001) (Table 3).

**Table 3:** Comparison of sociodemographic and clinical characteristics with CBI-24 and BIPQ score means.

Variables	BIPQ	95.0% CI		CBI-24	95.0% CI	
	Mean ± SD	Lower Bound	Upper Bound	Mean ± SD	Lower Bound	Upper Bound
Age	*r*: −0.129 *P*: .054	-0.256	0.002	*r*: −0.060 *P*: .375	−0.190	0.072
Dialysis vintage	*r*: 0.154 *P*: .021	0.023	0.280	*r*: 0.286 *P*: .000*	0.161	0.402
Gender						
Female	51.09 ± 5.24	−1.459	1.498	4.81 ± 0.47	−0.142	0.979
Male	51.07 ± 5.87			4.83 ± 0.44		
	*t*: 1.262 *P*: .979			*t*: 0.527 *P*: .715		
Cohen *d*	0.17			0.07		
Marital status						
Married	50.95 ± 5.93	−1.879	1.188	4.85 ± 0.46	−0.459	0.202
Single/widowed	51.29 ± 4.93			4.77 ± 0.43		
	*t*: 3.588 *P*: .657			*t*: 1.322 *P*: .216		
Cohen *d*	0.64			0.18		
Employment						
Yes	51.46 ± 5.84	−1.055	2.160	4.96 ± 0.43	0.746	0.330
No	50.91 ± 5.47			4.76 ± 0.45		
	*t*: 0.253 *P*: .499			*t*: 0.705 *P*: .002*		
Cohen *d*	0.04			0.10		
Education						
Basic literacy	50.22 ± 5.40	48.393	52.050	4.62 ± 0.52	4.444	4.796
Primary school	50.27 ± 5.41	49.125	51.419	4.81 ± 0.45	4.711	4.901
Middle school	51.93 ± 5.86	50.664	53.194	4.86 ± 0.42	4.772	4.955
University	53.14 ± 4.33	50.642	55.642	5.12 ± 0.24	4.980	5.263
	*F*: 2.229 *P*: .086			*F*: 4.938 *P*: .002*		
*η* ^2^	0.030			0.063		
Income						
Income < expenditure	50.29 ± 5.89	48.003	52.568	4.61 ± 0.49	4.422	4.804
Income = expenditure	51.24 ± 5.40	50.366	52.119	4.88 ± 0.43	4.811	4.951
Income > expenditure	51.02 ± 6.03	49.251	52.790	4.74 ± 0.46	4.608	4.877
	*F*: 0.348 *P*: .707			*F*: 5.123 *P*: .007**		
*η* ^2^	0.04			0.045		
Chronic illness						
No	54.12 ± 6.27	−1.919	1.671	4.84 ± 0.46	−0.112	0.179
Yes	50.70 ± 5.70			4.81 ± 0.45		
	*t*: 0.146 *P*: .127			*t*: 0.508 *P*: .652		
Cohen *d*	0.02			0.08		
Vascular access						
AVF	51.03 ± 5.69	50.147	51.914	4.88 ± 0.42	4.820	4.949
Permanent catheter	51.69 ± 5.10	50.252	53.119	4.70 ± 0.45	4.579	4.831
Temporary catheter	48.70 ± 5.93	44.460	52.939	4.30 ± 0.64	3.847	4.769
	*F*: 1.220 *P*: .297			*F*: 10.482 *P*: .000***		
*η* ^2^	0.011			0.087		
Help with care						
Yes	51.00 ± 5.62	−0.628	1.322	4.89 ± 0.44	0.251	0.262
No	51.15 ± 5.56			4.75 ± 0.46		
	*t*: 0.039 *P*: .838			*t*: 0.187 *P*: .018*		
Cohen *d*	0.01			0.03		

*r*: Pearson Correlation test; *F*: One-way ANOVA; *t*: Independent sample *t*-test.

ANOVA = analysis of variance, AVF = arteriovenous fistula, BIPQ = Brief Illness Perception Questionnaire, CBI-24 = Caring Behaviors Inventory-24, CI = confidence interval, HD = hemodialysis, SD = standard deviation.

* Significance between literate and middle school and between literate and university.

** Significance between income < expenditure and income = expenditure.

*** Significance between AVF and permanent and temporary, and between permanent and temporary.

*P* < .05; *P* < .01.

**Figure 1.**
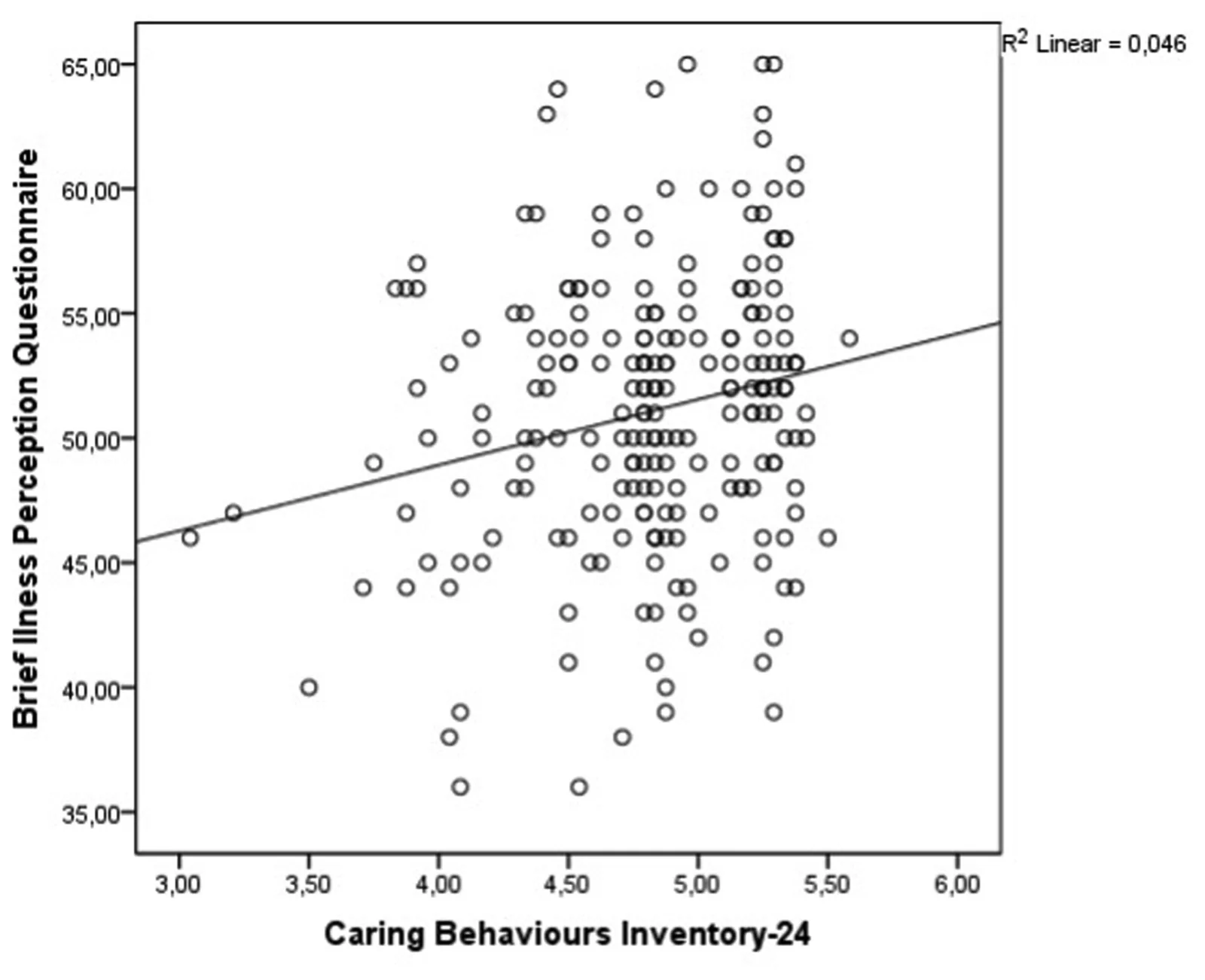
Relationship between CBI-24 and BIPQ scale scores. BIPQ = Brief Illness Perception Questionnaire, CBI-24 = Caring Behaviors Inventory-24, CI = confidence interval, SD = standard deviation.

The correlation between the CBI-24 and BIPQ total scores was examined, and a significant positive relationship was found (*r* = 0.214, 95% CI: 0.085–0.336, *P* = .001), indicating that as illness perception increased, caring behaviors also increased. When the subscales of the CBI-24 were examined, a significant negative correlation was found between the CBI-24 subscale of assurance and the BIPQ cognitive subscale (*r* = −0.152, 95% CI: −0.278–−0.021, *P* = .023), indicating that as cognitive illness perception increases, assurance decreases. A significant positive correlation was found between the knowledge and skill subscale of the CBI-24 and the BIPQ emotional subscale (*r* = 0.234, 95% CI: 0.106–0.355, *P* < .001), showing that as emotional illness perception increased, knowledge and skill scores also increased. A significant positive correlation was also found between the knowledge and skill subscales of the CBI-24 and the BIPQ cognitive subscale (*r* = 0.145, 95% CI: 0.014–0.271, *P* = .030), indicating that higher cognitive perception was associated with higher knowledge and skill scores (Table 4). Furthermore, significant positive correlations were observed between the CBI-24 subscales of being respectful and connectedness and the BIPQ emotional subscale (*r* = 0.192, 95% CI: 0.062–0.315, *P* = .004 and *r* = 0.275, 95% CI: 0.149–0.392, *P* < .001, respectively), indicating that emotional illness perception increased as the respectful and connectedness scores increased (Fig. 1).

**Table 4:** Correlation of BIPQ and CBI-24 score means.

	BIPQ									
	Emotional representations of illness				Cognitive representations of illness			Total score		
	Statistics *r* *P*		95.0% CI		Statistics *r* *P*	95.0% CI		Statistics *r* *P*	95.0% CI	
			Lower Bound	Upper Bound		Lower Bound	Upper Bound		Lower Bound	Upper Bound
CBI-24 total score	*r*	0.262**	0.135	0.380	0.062	−0.070	0.192	0.214**	0.085	0.336
	*P*	.000			.359			.001		
Assurance	*r*	0.009	−0.123	0.140	−0.152*	−0.278	−0.021	−0.100	−0.229	0.031
	*P*	.898			.023			.135		
Knowledge & skill	*r*	0.234**	0.106	0.355	0.145*	0.014	0.271	0.254**	0.127	0.373
	*P*	.000			.030			.000		
Being respectful	*r*	0.192**	0.062	0.315	0.063	−0.069	0.193	0.170*	0.039	0.294
	*P*	.004			.346			.011		
Connectedness	*r*	0.275**	0.149	0.392	0.118	−0.014	0.245	0.262**	0.135	0.380
	*P*	.000			.079			.000		

BIPQ = Brief Illness Perception Questionnaire, CBI-24 = Caring Behaviors Inventory-24, CI = confidence interval, HD = hemodialysis.

* *P* < .05.

** *P* < .01.

Linear regression analysis was performed to determine whether sociodemographic and clinical variables, together with illness perception dimensions, independently predicted the CBI-24 total scores (Table 5). The overall model was statistically significant (*F* = 8.878, *P* < .001), explaining 24.9% of the variance in CBI-24 scores (*R*^2^ = 0.249; adjusted *R*^2^ = 0.221). A higher educational level (*β* = 0.152, *B* = 0.083, 95% CI: 0.016–0.151, *P* = .016), longer hemodialysis vintage (*β* = 0.185, *B* = 0.020, 95% CI: 0.007–0.034, *P* = .003), and higher cognitive representations of illness (*β* = 0.161, *B* = 0.020, 95% CI: 0.005–0.035, *P* = .011) were independently associated with higher CBI-24 scores. In contrast, vascular access type (*β* = −0.271, *B* = −0.221, 95% CI: −0.319–−0.123, *P* < .001) and care support (*β* = −0.147, *B* = −0.133, 95% CI: −0.242–− 0.024, *P* = .017) were significant independent predictors of CBI-24 scores. Employment status, economic status, and emotional representations of illness were not significantly associated with CBI-24 scores (all *P* > .05) (Table 5).

**Table 5:** Multiple linear regression examining the effect of sociodemographic and clinical characteristics and BIPQ on CBI-24 scores.

	*ß*	Beta	*t*	*P*	95.0% CI	
					Lower Bound	Upper Bound
(Constant)	4477		13,265	.000	3812	5143
Employment	−.083	−.084	−1332	.184	−.206	.040
Education	.083	.152	2428	.016	.016	.151
Economy	−.050	−.064	−1037	.301	−.146	.045
Vascular access	−.221	−.271	−4444	.000	−.319	−.123
Help for care	−.133	−.147	−2397	.017	−.242	−.024
HD vintage	.020	.185	2963	.003	.007	.034
Emotional representations of illness	.006	.052	.872	.384	−.008	.020
Cognitive representations of illness	.020	.161	2575	.011	.005	.035

*r* = 0.499; *R*^2^ = 0.249; Adjusted *R*^2^ = 0.221.

*F* = 8.878; *P* = .000.

CBI-24 = Caring Behaviors Inventory-24, HD = hemodialysis.

## 4. Discussion

Our study found that the scores for the CBI-24 subscales were as follows: assurance, 4.65 ± 0.53; knowledge and skills, 4.87 ± 0.82; being respectful, 4.86 ± 0.77; connectedness, 4.98 ± 0.78; and total score, 4.82 ± 0.45. In a study by Cerit and Coşkun with 114 patients, examining the perceptions of patients and nurses relating to care quality, it was found that the patients’ CBI-24 total score was 4.94 ± 0.84, and that the highest subscale score was knowledge and skill, and the lowest was connectedness.^[18]^ In the same study, the mean subscale scores were 4.98 ± 0.93 for assurance, 5.13 ± 0.77 for knowledge and skills, 4.87 ± 0.90 for being respectful, and 4.75 ± 0.94 for connectedness. The knowledge and skills subscale scores were higher than those observed in this study. This difference may be related to differences in clinical settings, patient characteristics, and the organization of nursing care across institutions. In a study by Eroğlu (2018) evaluating the caring behavior of nurses in relation to 108 patients in the emergency service, it was found that the CBI-24 total score was 5.94 ± 0.10. The subscale scores were 5.96 ± 0.15 for assurance, 5.97 ± 0.07 for knowledge and skills, 5.92 ± 0.15 for being respectful, and 5.89 ± 0.20 for connectedness. The substantially higher CBI-24 scores reported in the emergency department setting may reflect differences in patient expectations, care context, and the acute nature of nurse–patient interactions. In the present study, the highest subscale score was for connectedness, which differs from previous findings. This may be related to the extended contact time between nurses and patients during hemodialysis sessions, as well as the structured education and counseling practices in our center, which foster stronger nurse–patient bonds.^[19]^ The connectedness subscale assesses nurses’ behaviors related to informing and supporting patients, allocating sufficient time to them, and understanding and planning care together. Hemodialysis sessions take place approximately 3 times a week for 4 hours. During this period, the nurse is a health professional who spends the most time with the patient. During this time, it is essential to inform the patient about topics such as diet and fluid restriction, potential complications, and lifestyle adjustments, and to dedicate sufficient time to the patient. The positive association between longer dialysis duration and higher caring behavior scores may also reflect the development of stronger therapeutic relationships and increased patient familiarity with nursing routines over time, which can enhance trust and engagement. It is believed that involving patients in planning their care leads to improvements in patient outcomes, a positive perception of nursing care quality, and, ultimately, satisfaction with health services.^[9]^ These findings emphasize that the interpersonal aspects of nursing care may be particularly valued by patients receiving long-term hemodialysis.

In our study, the mean BIPQ total score was 51.08 ± 5.58, the emotional subscale score was 32.77 ± 3.65, and the cognitive subscale score was 18.31 ± 3.88. These findings suggest that hemodialysis patients have a moderate level of illness perception. In a study by Özer et al (2021) involving 200 hemodialysis patients who used fatalism in medicine and the revised BIPQ, the total mean score was found to be 29.7 ± 10.97. Among the BIPQ subscales, the highest mean score was observed for the consequences dimension (8.02 ± 1.81).^[20]^ In a study by Tuncel (2024) involving 240 hemodialysis patients, the results showed that the BIPQ total score was 62.78 ± 7.86. Among the BIPQ subscales, the treatment control dimension had the highest mean score (9.29 ± 1.37).^[21]^ In the Turkish cultural context, where family involvement in patient care is strong and interpersonal relationships are highly valued, patients may place particular importance on the relational aspects of care, such as connectedness and emotional support.

Our study revealed that as the number of years of dialysis increased, caring behaviors in patients also increased. Hemodialysis nurses play a crucial role in the unit by facilitating care, educating patients, and providing psychosocial counseling. Nurses are in a better position than other healthcare professionals to have a positive impact on caring behaviors by developing therapeutic relationships in patient care.^[22]^ However, this relationship may be bidirectional; stronger caring behaviors could improve illness perception, while patients with more positive illness perceptions may also be more receptive to nursing care. Therefore, a patient who has been receiving hemodialysis for many years benefits the most from nursing care. This finding may reflect the gradual development of trust and familiarity between patients and nurses over time, allowing patients to recognize and appreciate caring behaviors more clearly.

In this study, it was found that care dependency behaviors of working patients were higher than those of nonworking patients. The literature shows that in chronically ill individuals, financial losses related to a reduction in productivity result from an inability to continue working because of illness. This has a negative impact on both physical and psychological health.^[23]^ Better economic status may reduce financial stress, allowing patients to focus more on treatment and self-care, which could partly explain the positive association with higher scores on caring behavior. Therefore, having a job has a positive impact on a patient sense of purpose in life. This positive attitude is reflected in caring behaviors.

It has been reported that chronic renal failure and mortality rates are higher at lower educational levels. Patients’ health protection, development, and maintenance levels were also higher at higher education levels. Patients can easily understand and implement the education provided by nurses, as well as the quality of care they receive. Thus, they face less damage from dialysis symptoms, and their quality of life is higher.^[24]^ It was also observed in the findings of our study that, as supported by the literature, caring behaviors increased with increasing education levels.

Economic status is crucial for maintaining a stable living situation and covering essential expenses including food, shelter, and healthcare. The study findings revealed that patients with sufficient income to meet their expenses exhibited better caregiving behaviors. Ng et al (2021) reported in a systematic review on financial difficulties in dialysis patients that a poor financial situation, resulting from a low-income level, had a negative impact on these patients.^[22]^ Our findings support the literature, which concludes that economic status, particularly having sufficient funds for health expenses, has a positive effect on caring behaviors.

AVFs are one of the most commonly used permanent vascular access routes for hemodialysis treatment. They have better blood flow than other vascular accesses and have a lower risk of infection and thrombosis. Additionally, a lower rate of morbidity and mortality was observed compared to other vascular access routes.^[25,26]^ At the same time, self-care behaviors are important for protecting against AVF.^[25]^ It was determined in the findings of our study that patients with an AVF had better care behaviors than those with a temporary catheter. Self-care behaviors in patients have improved as a result of good care behaviors, which, in turn, contribute to the preservation of AVF and indirectly to dialysis competence.

In our study, it was found that the care behavior scores of patients who felt the need for help with caring were higher. A 2023 study, conducted with 160 patients with heart failure to determine illness perception and care-seeking intention, reported that patients who perceived the effects of treatment sought care at an earlier stage. Therefore, educating patients primarily on the benefits of treatment may increase their intention to seek medical assistance.^[27]^ Outside the hospital, a family member is generally concerned with the care of individuals. This is also the case in the society where the study was conducted. In the literature, family members typically provide the majority of care for hemodialysis patients, and it has been reported that they receive the most social support from family members.^[28–30]^ In our study, we found that the care behavior scores of patients who felt the need for help with care were higher. Hemodialysis patients receive support from caregivers in managing the numerous complications they experience either during or after dialysis sessions, and this has been observed to have a positive effect on care behaviors.

A final observation of the present study was a statistically significant positive association between nursing caring behavior scores and illness perception scores, suggesting that patients who perceived higher levels of nursing care also reported more adaptive illness representations. Positive illness perception in hemodialysis patients can help individuals cope more effectively with their disease and support better adaptation to treatment processes. In contrast, negative illness perceptions may increase resistance to treatment and adversely affect psychological health.^[31]^ In particular, as the duration of the illness increases, situations such as the inability to adapt to the treatment regime (being dependent on the hemodialysis machine and the center, diet, liquid restrictions, etc) can have negative psychological effects on the individual. Therefore, illness perceptions may be negatively affected.^[5]^ Nursing care in hemodialysis is effective in improving patient adherence to dialysis. Various interventions, including appropriate education and counseling, fluid and dietary management, medication management, psychosocial support, and palliative care, contribute positively to an individual’s perception of their illness. Education and counseling help patients and their families to gain knowledge about the treatment process, thereby increasing treatment adherence and reducing the risk of complications. Fluid and dietary management reduces the risk of complications by regulating fluid intake and diet. Medication management is essential to control hypertension, anemia, and mineral metabolism disorders. Psychosocial support helps patients cope with the psychological and social challenges that they encounter during the treatment process.^[31]^ Nevertheless, despite statistical adjustments, residual confounding by unmeasured factors such as personality traits or unrecorded comorbidities cannot be completely ruled out. Additionally, dialysis patients’ dependence on dialysis and complications of dialysis treatment increase their illness perceptions, which is a natural consequence. At the same time, it was observed that patients were aware of their own care needs, as well as their perception of the treatment and care provided to them, and their care behaviors at each dialysis session also increased. Negative thoughts about the illness can be reduced by using an individualized and holistic nursing care approach. Thus, adherence to the treatment regime can be further increased. Furthermore, patients who perceive greater support from nurses may feel more confident in managing their illness, which may positively influence both emotional adjustment and engagement with treatment.

When the subscales of the CBI-24 were examined, a significant negative correlation was found between the Assurance dimension and the Cognitive dimension of the BIPQ (*r* = −0.152, *P* = .023), indicating that patients with more negative cognitive perceptions of their illness reported lower levels of nursing assurance. According to Leventhal Common-Sense Model, patients develop cognitive and emotional representations of their illness that influence how they cope with their condition and respond to treatment. Poor illness coherence and perceived control have been associated with less adaptive illness perceptions and poorer health outcomes.^[32]^ Our findings are consistent with this model, suggesting that patients who perceive their illness as less understandable or less controllable may feel less reassured by nursing care. This highlights the importance of individualized education and counseling to strengthen patients’ understanding of their illnesses and enhance their confidence in nursing support.^[6,33]^

Positive correlations were also observed between the knowledge and skill dimension and both the emotional and cognitive dimensions of illness perception, suggesting that patients who perceive nurses as knowledgeable may better understand and adapt to their illness. Likewise, the associations between the being respectful and connectedness dimensions and emotional illness representations emphasize the value of respectful communication and therapeutic relationships in helping patients cope with the emotional challenges of long-term hemodialysis.

Finally, the regression analysis showed that illness perception remained an independent predictor of nursing care behaviors after adjusting for sociodemographic and clinical characteristics, indicating that illness perception contributes to patients’ experiences of nursing care beyond clinical factors alone.

### 4.1. Limitations

This study has several limitations that should be acknowledged. First, the cross-sectional and correlational design precludes causal inferences regarding the relationship between illness perception and caregiving behavior; longitudinal studies are required to track changes in these variables over time. Second, the data relied exclusively on patient self-reports, introducing potential social desirability bias. This risk may have been heightened as patients evaluated nursing care while actively receiving treatment in the dialysis unit, potentially leading to overly favorable ratings. Third, although standardized protocols were strictly followed, the questionnaires were administered directly by the research team, meaning interviewer bias cannot be entirely ruled out. Future research employing longitudinal designs, fully anonymized data collection, and independent or blinded interviewers will be crucial to mitigate these limitations.

### 4.2. Implications for clinical practice

This study contributes to nursing knowledge by demonstrating that illness perception is associated not only with overall perceptions of nursing care behaviors but also with specific dimensions of caring among patients receiving maintenance hemodialysis. These findings highlight the importance of addressing patients’ perceptions of illness as part of routine nursing care. Future research should evaluate whether interventions targeting illness understanding, perceived control, and patient education can improve specific dimensions of nursing care behaviors and strengthen therapeutic nurse–patient relationships across different dialysis settings.

## 5. Conclusion

This study revealed that a longer duration of illness was associated with a negative impact on illness perception among hemodialysis patients. Significant positive correlations were observed between the knowledge and skill subscale and the emotional and cognitive subscales, as well as between both the being respectful and connectedness subscales and the emotional subscale. These findings emphasize the necessity of individualized and holistic nursing approaches for nephrology nurses to improve patients’ illness perceptions and enhance treatment adherence. However, given that these results are limited to the specific hemodialysis sample studied, they should not be generalized to broader patient populations.

## Acknowledgements

We thank all the hemodialysis patients.

## Author contributions

**Conceptualization:** Özlem Bulantekin Duzalan, Elif Bulbul, Selda Selimoglu Namoglu.

**Data curation:** Elif Bulbul, Selda Selimoglu Namoglu

**Formal analysis:** Özlem Bulantekin Duzalan, Elif Bulbul.

**Methodology:** Özlem Bulantekin Duzalan, Elif Bulbul.

**Supervision:** Özlem Bulantekin Duzalan, Selda Selimoglu Namoglu.

**Writing** – **original draft:** Özlem Bulantekin Duzalan, Elif Bulbul.

**Writing** – **review & editing:** Özlem Bulantekin Duzalan, Elif Bulbul.
